# A viral-specific CD4^+^ T cell response protects female mice from Coxsackievirus B3 infection

**DOI:** 10.3389/fimmu.2023.1327384

**Published:** 2024-01-11

**Authors:** Aryamav Pattnaik, Adeeba H. Dhalech, Stephanie A. Condotta, Caleb Corn, Martin J. Richer, Laura M. Snell, Christopher M. Robinson

**Affiliations:** Department of Microbiology and Immunology, Indiana University School of Medicine, Indianapolis, IN, United States

**Keywords:** Coxsackievirus B3, Enteric virus, CD4 T cells, T cells, viral clearance, sex differences

## Abstract

**Background:**

Biological sex plays an integral role in the immune response to various pathogens. The underlying basis for these sex differences is still not well defined. Here, we show that Coxsackievirus B3 (CVB3) induces a viral-specific CD4+ T cell response that can protect female mice from mortality.

**Methods:**

We inoculated C57BL/6 Ifnar^-/-^ mice with CVB3. We investigated the T cell response in the spleen and mesenteric lymph nodes in male and female mice following infection.

**Results:**

We found that CVB3 can induce expansion of CD62Llo CD4+ T cells in the mesenteric lymph node and spleen of female but not male mice as early as 5 days post-inoculation, indicative of activation. Using a recombinant CVB3 virus expressing a model CD4+ T cell epitope, we found that this response is due to viral antigen and not bystander activation. Finally, the depletion of CD4+ T cells before infection increased mortality in female mice, indicating that CD4+ T cells play a protective role against CVB3 in our model.

**Conclusions:**

Overall, these data demonstrated that CVB3 can induce an early CD4 response in female but not male mice and further emphasize how sex differences in immune responses to pathogens affect disease.

## 1 Introduction

Biological sex is critical in the immune response to various pathogens (1, 2). Generally, women exhibit a more robust immune response to infections than men, and women often develop higher antibody responses to vaccines (3). This sex bias is likely due to differences in the number and activation of immune cells. For example, women have higher CD4^+^ to CD8^+^ T cell ratios than men (1). Also, T cells from females have enriched expression of genes involved in T cell activation (4). Furthermore, women exhibit stronger type I interferon responses, and female antigen-presenting cells are more efficient in presenting peptides than their male counterparts (5–7). Therefore, females may be inherently primed to mount more robust immune responses that limit pathogen replication and pathogenesis better than males. However, this comes at a cost, as females frequently experience more severe infection symptoms, more adverse vaccine reactions, and are more vulnerable to autoimmune disorders (2). Unfortunately, the mechanism dictating the sex bias in immunity remains elusive. Thus, knowing how sex impacts immune responses to infections and vaccines is crucial for therapeutic development and to aid in vaccine strategies to target both men and women.

Enteroviruses continue to pose a substantial global public health issue (8–11). These viruses are a group of positive-sense stranded RNA viruses classified in the Picornavirus family. Among enteroviruses, Coxsackievirus B3 (CVB3) is commonly isolated during surveillance and causes viral myocarditis and aseptic meningitis (12, 13). CVB3 is transmitted through the fecal-oral route and initiates infection in the intestine. Sex is known to be a pivotal contributor to CVB3 infections in humans. Men are two to three times more likely to develop myocarditis than women (14). This disparity is also mimicked in mouse models of CVB3 infection. Immune differences between males and females have been implicated in the pathology of CVB3-induced myocarditis; however, the mechanisms leading to these events are still largely unclear.

Data from C57BL/6 mouse models show that acute CVB3-induced myocarditis is characterized by inflammatory infiltration of immune cells, including CD4^+^ and CD8^+^ T cells (15, 16). Here, T cells likely contribute to viral clearance; however, other mouse models indicate that T cells can also contribute to CVB3-induced disease (17–20). In infection of Balb/c mice, CVB3 induces autoimmune-mediated myocarditis. In this model, differences in the CD4^+^ T helper (Th) 1 and Th2 response following infection contribute to the sex bias in myocarditis. Following CVB3 infection, males mount a predominantly Th1, which enhances acute inflammation and myocarditis (18, 20). In contrast, females skew towards a Th2 response that can limit inflammation in the heart. The mechanism for this difference is still unclear, but sex hormones can contribute to the Th1/Th2 balance following infection (21). Moreover, γδ T cells and CD4^+^ T regulatory cells (Tregs) can also promote or limit disease (22–25). Overall, these data indicate that T cells largely contribute to disease, yet how CD4^+^ T cells are activated in acute CVB3 infection and how these T cells contribute to cell-mediated viral clearance is incompletely understood.

Research from our lab and many others have shown that sex hormones influence the pathogenesis of CVB3 in mice (21, 26–29). Using an oral inoculation mouse model, our lab has demonstrated that gonadectomy impacts intestinal CVB3 replication and dissemination in male and female mice (26, 27). Castration of male mice also completely protected males from mortality following CVB3 infection, and exogenous testosterone treatment restored lethality. This suggests that testosterone plays a vital role in the mortality of male mice infected with CVB3. However, the impact of testosterone is complex since exogenous testosterone treatment in female mice did not increase mortality. These data indicate other immune cells may protect female mice from CVB3 infection. In support of this, we recently identified a sex difference in the T cell response to CVB3. We demonstrated that activated, viral-specific CD8^+^ T cells in female but not male mice expand and protect against CVB3 infection (30). Similarly, we found that CD4^+^ T cells expand in a sex-dependent manner. Here, we sought to explore further the CD4^+^ T cell response to CVB3 infection in male and female mice. Our data indicate that CD4^+^ T cells are activated in female but not male mice as early as 5 post-inoculation (dpi), and viral-specific CD4^+^ T cells expand in females but not males. Finally, we show that CD4^+^ T cells are protective against CVB3 infections in female mice. These data add to the growing body of evidence highlighting the existence of sex-specific immune regulation in response to viral infections.

## 2 Materials and methods

### 2.1 Cells and virus

HeLa cells were cultivated in Dulbecco’s modified Eagle’s medium (DMEM) supplemented with 10% calf serum and 1% penicillin-streptomycin. The cells were maintained at 37˚C in an environment containing 5% CO2. The infectious clones CVB3-Nancy and recombinant CVB3-H3 were obtained from Marco Vignuzzi at the Pasteur Institute in Paris, France. and transfected in HeLa cells as previously described (26). A standard plaque assay with HeLa cells was used to quantify the virus. The rCVB3.6 was graciously provided to us by Lindsay Whitton and Taishi Kimura (Scripps Research Institute in La Jolla, California) and passaged in HeLa cells.

### 2.2 Mouse experiments

C57BL/6, *PVR^+/+^*, *Ifnar-/-* mice were obtained from S. Koike in Tokyo, Japan (31, 32). Age-matched mice, ranging in age from 8 to 14 weeks at the time of infection, were used for all experiments. Male and female mice were orally inoculated with 5x10^7^ PFUs or intraperitoneally inoculated with 1x10^4^ PFUs of CVB3-Nancy. For rCVB3.6 experiments, mice were intraperitoneally inoculated with 1x10^4^ PFU of CVB3-H3 as control or 5x10^7^-1x10^8^ PFU of rCVB3.6. Data from the mouse investigations were combined from two to three experiments, with each group consisting of at least three mice per study.

### 2.3 SMARTA T cells and adoptive transfer

Transgenic T cells with TCRs specific to LCMV GP_61-80_ peptide were isolated from SMARTA mice, which have been previously characterized and were bred on a CD45.1 background (33). SMARTA T cells (CD45.1) were adoptively transferred into our *Ifnar^-/-^* (CD45.2) mouse model i.v. via the retro-orbital sinus one day before infection. Female *Ifnar^-/-^* mice were then ip inoculated with 10^8^ PFU of rCVB3.6 or 10^4^ PFU of wild-type CVB3 (wtCVB3) as a control. As a control for gating GP_66_-specific CD4 T cells, we also infected female *Ifnar^-/-^* mice with LCMV (data not shown). The spleen was harvested from infected mice at 5dpi, and virus-specific SMARTA CD4 T cells were analyzed by flow cytometry by expression of CD45.1.

### 2.4 Flow cytometry analysis

At indicated time points post-infection, the spleen and mesenteric lymph nodes from male and female mice were collected and mechanically disrupted to obtain a single-cell suspension. RBC lysis buffer from BioLegend (catalog # 420301) was used to remove erythrocytes. Afterward, the cells were washed and incubated with TruStain fcX (CD16/CD32, Clone 93, BioLegend, catalog # 101320) to prevent non-specific binding, and the immune cells of interest were then stained with appropriate surface antibodies. The samples were subjected to analysis using a BD LSRFortessa flow cytometer in combination with FlowJo software (BD Biosciences). The following mouse antibodies, in various fluorochrome combinations, were utilized for the staining: CD4 (clone GK1.5, BioLegend, catalog #100412, #100406), CD8a (clone 53-6.7, BioLegend, catalog, #100707 #100711), CD11a (clone M17/4, BioLegend, catalog # 101106), CD62L (clone MEL-14, BioLegend, catalog #104438), CD49d (clone R1-2, BioLegend, catalog #103618).

### 2.5 CD4^+^ T cell depletion

Two days before infection, CD4^+^ T cells were depleted in male and female mice by intraperitoneally injecting mice with 200 μg of an anti-CD4 antibody (anti-mouse CD4 clone: GK1.5, BioXcell, catalog # BE0003-1). A group of mice receiving an intraperitoneal injection of an isotype control antibody (rat IgG2b LTF-2, BioXcell, catalog # BE0090) was used as a control. The following day, a tail vein blood sample was collected from each mouse. The blood samples were then stained and analyzed by flow cytometry to confirm the successful depletion of CD4^+^ T cells.

### 2.6 Statistical analysis

Comparisons between the control and study groups were analyzed using either an unpaired t-test or a one-way analysis of variance (ANOVA), depending on the experimental design. To visualize the variability, error bars in the figures were plotted to represent the standard errors of the means. A significance level of p < 0.05 was used to determine if there were any meaningful differences between the groups. All statistical analyses and graphs were generated using GraphPad Prism 10 (GraphPad Software, La Jolla, CA), ensuring accurate and comprehensive data representation.

## 3 Results

### 3.1 Antigen-experienced CD4^+^ T cells expand in the mesenteric lymph nodes of CVB3-infected female but not male mice

**Figure 1:**
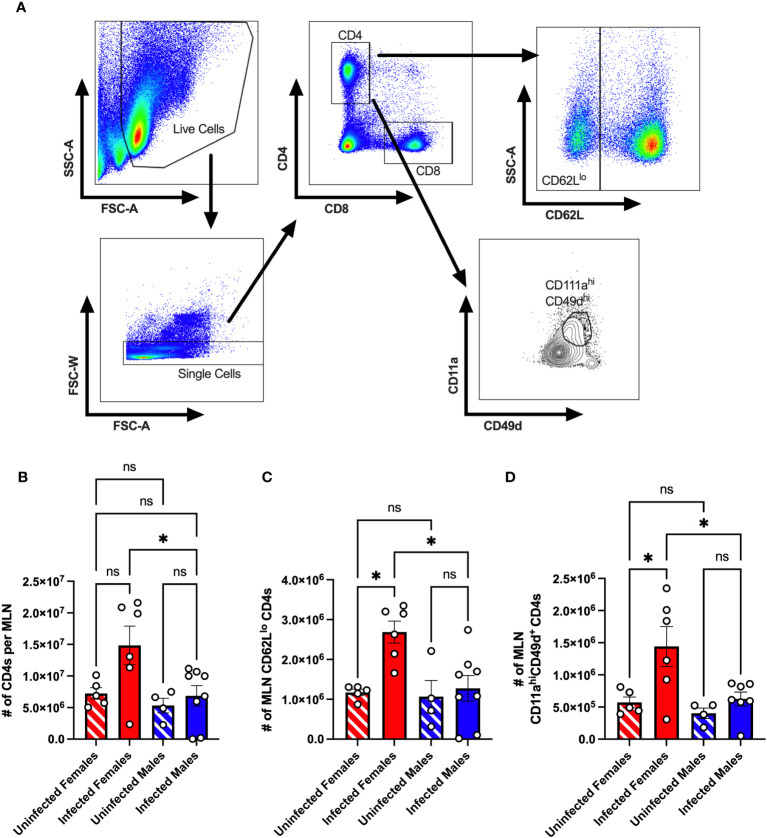
The CD4^+^ T cell response in the mesenteric lymph node of CVB3-infected *Ifnar^-/-^* mice. Male and female *Ifnar^-/-^* mice were orally inoculated with 5x10^7^ PFUs of CVB3. The mesenteric lymph nodes (MLN) were harvested at 5dpi and processed for analysis by flow cytometry. **(A)** Representative gating strategy for CD4^+^ T cells, CD62L^lo^ CD4^+^ T cells, and CD11a^hi^CD49d^+^ CD4 T cells. **(B)** The number of MLN CD4^+^ T cells in male and female mice 5dpi. **(C)** The number of CD62L^lo^ CD4^+^ T cells in the MLNs at 5dpi. **(D)** The number of MLN CD11a^hi^CD49d^+^ CD4 T cells. Data points represent individual mice. Data are from at least two independent experiments. *p<0.05, One-way ANOVA. ns, not significant.

We previously found that following oral inoculation, CVB3 induces a sex-dependent expansion of T cells in the spleen (30). However, since CVB3 initiates infection in the intestine, we examined the CD4^+^ T cell response in the local lymph node following infection. Male and female *Ifnar^-/-^* mice were orally inoculated with 5x10^7^ PFUs of CVB3, and the mesenteric lymph nodes (MLNs) were harvested at 5dpi. We chose to examine immune cells at 5dpi because we have previously shown that mortality in male mice typically begins at this time point (26, 27). Following CVB3 infection, we found that the number of CD4^+^ T cells showed a trending increase in the infected female mice compared to uninfected female mice (**Figures 1A**, **1B**). However, this increase was not statistically significant (p=0.0816). In contrast, we observed a significant increase in the number of CD4^+^ T cells in infected female *Ifnar^-/-^* mice compared to infected male mice (**Figure 1B**). Next, we assessed the CD4^+^ T cell activation by the expression of CD62L. Naïve T cells are CD62L^hi^ and activated T cells differentiate into effector subtypes during acute infections. During this effector phase, CD62L is downregulated (34–36). Following oral inoculation, we found a significant increase in the number of CD62L^lo^ CD4^+^ T cells in infected female mice compared to uninfected female mice and infected male mice (**Figure 1C**). Moreover, this increase was sex-dependent and not observed between infected and uninfected male mice.

Next, the downregulation of CD62L on CD4^+^ T cells can occur through bystander activation rather than direct antigen-specific engagement of the T cell receptor (37). Previous studies have established that antigen-experienced T cells can be followed regardless of their specificity using surrogate markers (34, 38–42). To investigate if CVB3-induced virus-specific CD4^+^ T cells in the mesenteric lymph nodes, we measured the expression of CD11a and CD49d on CD4^+^ T cells as a measure of antigen-experienced CD4^+^ T cells (**Figure 1A**). We found a significant increase in the number of CD11a^hi^CD49d^+^ CD4^+^ T cells in infected females compared to uninfected females (**Figure 1D**). In contrast, no difference in the number of CD11a^hi^CD49d^+^ CD4^+^ T cells between uninfected and infected males was observed. Moreover, the number of CD11a^hi^CD49d^+^ CD4^+^ T cells in infected female mice was significantly higher than in infected male mice. Taken together, these data indicate that CVB3 induces activated, antigen-experienced CD4^+^ cells at 5dpi in mesenteric lymph nodes of female but not male *Ifnar^-/-^* mice.

### 3.2 Sex-dependent activation of CD4^+^ T cells in the spleen occurs as early as 5 dpi in CVB3-infected mice

**Figure 2:**
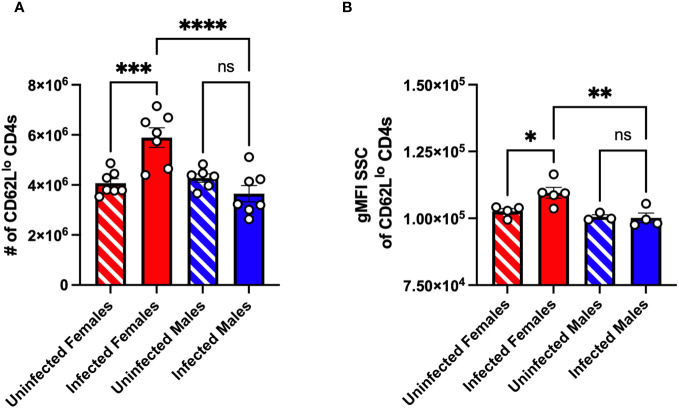
CVB3 induces early expansion of activated splenic CD4^+^ T cells in female *Ifnar^-/-^* mice. **(A)** The number of CD62L^lo^ CD4^+^ T cells in the spleen 5dpi in *Ifnar^-/-^* mice. **(B)** Representative of the gMFI SSC of CD62L^lo^ CD4^+^ T cells from two independent experiments. Data points represent individual mice. Data are from two independent experiments.*p<0.05, **p<0.01, ***p<0.001, ****p<0.0001 One-way ANOVA.

Previous studies in C57BL/6 mice indicated a limited T cell response following CVB3 infection (17, 43, 44). However, these studies were conducted exclusively using male mice. In contrast, we previously found that splenic CD4^+^ and CD8^+^ T cells expand at 5dpi in orally CVB3-infected female *Ifnar^-/-^* mice but not infected male *Ifnar^-/-^* mice (30). Also, in contrast to antigen-experienced CD8^+^ T cells that increase as early as 5 dpi, we found that splenic antigen-experienced CD4^+^ T cells from female mice do not increase until 15 dpi (30). However, we hypothesized that splenic CD4^+^ T cells may be undergoing signs of early activation even though the upregulation of the cell surface expression of the surrogate makers, CD11a and CD49d, due to recognition of antigen (38, 42, 45) had not occurred. Therefore, to test this hypothesis, we assessed CD4^+^ T cell activation in the spleen by the expression of CD62L. Male and female *Ifnar^-/-^* mice were orally inoculated with 5x10^7^ PFUs of CVB3, and the spleen from infected and uninfected mice was harvested at 5 dpi. Following CVB3 inoculation, we observed a significant increase in the number of CD62L^lo^ CD4^+^ T cells in infected female mice compared to infected male mice at 5dpi (**Figure 2A**). Further, infected female mice had significantly higher numbers of CD62L^lo^ CD4^+^ T cells than uninfected female mice. In contrast, no difference was observed in the number of CD62L^lo^ CD4^+^ T cells between uninfected and infected male mice. Next, to confirm that the CD4^+^ T cells were becoming activated in female mice but not male mice, we examined if the CD4^+^ T cells were undergoing blast transformation. Within a few hours of antigen activation, CD4^+^ T cells can undergo blast transformation, which results in increased granularity as they develop into mature effector cells (46, 47). To measure changes in granularity, we assessed the mean fluorescence intensity (gMFI) for the side scatter area (SSC) of the CD62L^lo^ CD4^+^ T cell population. We found that CD62L^lo^ CD4^+^ T cells from infected female mice had a significant increase in granularity, as measured by the SSC, compared to CD62L^lo^ CD4^+^ T cells from uninfected female mice (**Figure 2B**). Overall, these data indicate that CVB3 induces early activation of CD4^+^ T cells at 5 dpi in female mice but not male mice.

### 3.3 CVB3 induces viral-specific splenic CD4^+^ T cells as early as five days post-inoculation

**Figure 3:**
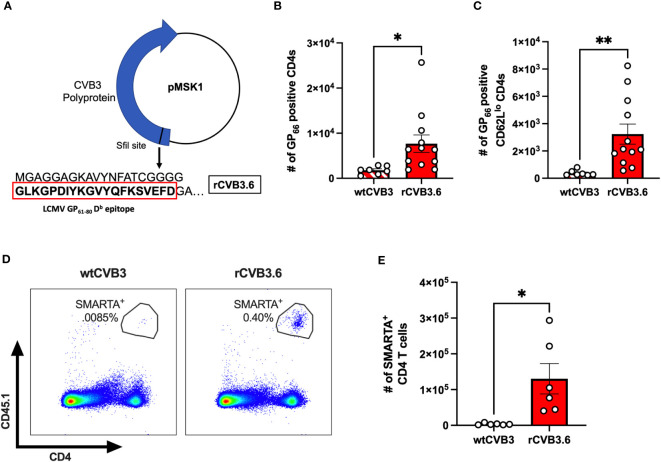
CVB3 induces a virus-specific expansion of CD4^+^ T cells in female mice. **(A)** The rCVB3.6 amino acid sequence encoding the LCMV GP_61-80_ CD4^+^ T cell epitope [adapted from (17)]. In this recombinant virus, the GP_61-80_ epitope is inserted upstream of the open reading frame encoding the polyprotein, separated by a poly-glycine linker and an artificial 3Cpro/3CDpro cleavage site. **(B)** The number of splenic CD4^+^ T cells detected with H2-D^b^ GP_66_ tetramer in female *Ifnar^-/-^* mice 15dpi. **(C)** The number of H2-D^b^ GP_66_ tetramer positive splenic CD62L^lo^ CD4^+^ T cells. **(D)** Representative gating strategy for SMARTA^+^ T cells, based on CD45.1 expression, following infection with wtCVB3 or rCVB3.6. **(E)** The number of splenic SMARTA^+^ T cells in female mice infected with wtCVB3 or rCVB3.6. All data are from two independent experiments and are shown as mean ± SEM. *p<0.05; **p<0.01, unpaired t-test.

Unfortunately, few studies have identified immunodominant T cell epitopes for CVB3. Therefore, to determine if the CD4^+^ T cell response was viral-specific, we used a recombinant CVB3 (rCVB3.6) that encodes a well-characterized CD4^+^ T cell epitope from lymphocytic choriomeningitis virus (LCMV) (**Figure 3A**). This recombinant CVB3, while attenuated *in vivo*, still leads to productive infection, generating high tissue titers, and is cleared similarly to wild-type CVB3 (17, 43, 44). We ip inoculated female *Ifnar^-/-^* mice with rCVB3.6 or wild-type CVB3 (wtCVB3) as a control. The spleen was harvested from infected mice at 15 dpi, and virus-specific CD4^+^ T cells were analyzed by flow cytometry using an H2-D^b^ GP_66_ tetramer. We initially chose to study CD4^+^ T cells at 15 dpi, based on our previous data measuring the expansion of antigen-experienced CD4^+^ T cells (30). At 15 dpi, we observed a significant increase in the number of GP_66_-specific CD4^+^ T cells in the spleen from female mice infected with the rCVB3.6 virus compared to female mice infected with wtCVB3 (**Figure 3B**). We observed a significant increase in the number of GP_66_^+^CD62L^lo^ CD4^+^ T cells from female mice inoculated with rCVB3.6 compared to wtCVB3 infected mice (**Figure 3C**). Overall, these data indicate that CVB3 drives the expansion of activated, virus-specific CD4^+^ T cells at 15 dpi in female mice.

Next, since we observed markers of activation on CD4^+^ T cells in the spleen as early as 5 dpi, we reasoned that the expression of surrogate markers may not detect early virus-specific CD4s. Therefore, we adoptively transferred SMARTA T cells into our mouse model to enhance our sensitivity. T cells from SMARTA mice have a transgenic T cell receptor for the LCMV GP_61-80_ T cell epitope encoded in the rCVB3.6 (48, 49). Following the adoptive transfer of SMARTA T cells, we ip inoculated mice with either rCVB3.6 or wtCVB3 and harvested the spleen 5 dpi. We found a significant increase in SMARTA CD4 T cells in the spleen of female mice inoculated with rCVB3.6 compared to wtCVB3 (**Figures 3D, E**). These data indicate that virus-specific CD4^+^ T cells are expanding in the spleen of infected mice as early as 5 dpi in ip inoculated female mice.

### 3.4 CD4^+^ T cells protect female mice but not male mice from CVB3-induced lethality

**Figure 4:**
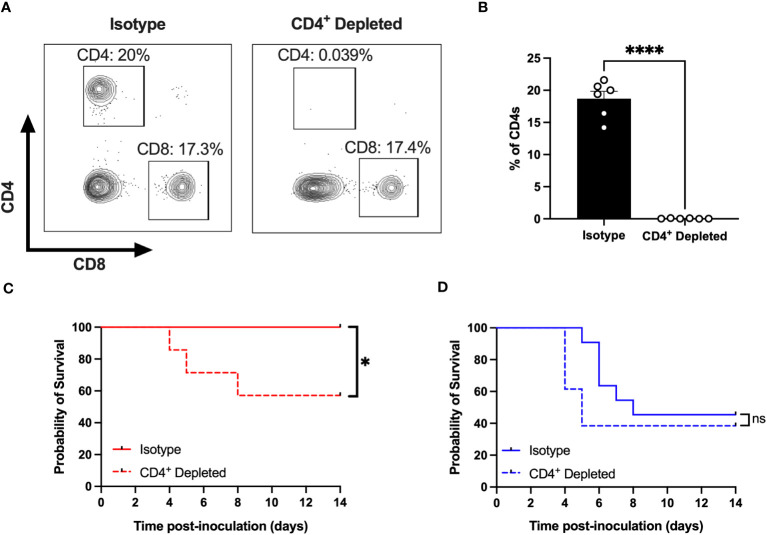
CD4^+^ T cells protect females from CVB3-induced lethality. **(A)** Representative flow cytometry plots of T cells gated on CD4^+^ and CD8^+^ T cell expression in female *Ifnar^-/-^* mice treated with an anti-CD4 antibody or isotype control. **(B)** The representative frequency of CD4^+^ T cells in female *Ifnar^-/-^* mice treated with an anti-CD4 antibody or isotype control from two independent experiments. ****p<0.0001, unpaired t-test. **(C)** Survival of CVB3-infected female mice treated with anti-CD4 or isotype control antibody. **(D)** Survival of CVB3-infected male mice treated with anti-CD4 or isotype control antibody. Survival data are collected from three independent experiments. n=11-14 mice per group. *p<0.05, Log-rank (Mantel-Cox) test. ns, not significant.

We have previously shown that female *Ifnar^-/-^* mice are protected against CVB3-induced mortality (26, 27). Since CD4^+^ T cells expand in female mice following infection, we hypothesized that these T cells might offer protection against CVB3-induced lethality. To test this hypothesis, we depleted CD4^+^ T cells with a monoclonal antibody before CVB3 inoculation (**Figures 4A, B**). Following CVB3 infection, we observed a significant increase in mortality in female mice depleted of CD4^+^ T cells compared to female mice treated with an isotype control (**Figure 4C**). In contrast to females, depletion of CD4^+^ T cells in males did not significantly enhance mortality (**Figure 4D**). These data demonstrate that CD4^+^ T cells play a significant role in protection against CVB3 in female mice.

## 4 Discussion

Previous research from our laboratory and others has demonstrated a strong sex bias in CVB3 pathogenesis (27, 30). The mechanisms dictating this sex bias are incompletely understood. Here, we found significantly more activated, viral-specific CD4^+^ T cells in infected female mice compared to male mice in the mesenteric lymph node and spleen as early as 5dpi. Moreover, the depletion of these CD4^+^ T cells protected female mice. These data provide evidence that activation and expansion of CD4^+^ T cells play a crucial role in limiting CVB3 pathogenesis and further demonstrate the importance that sex plays in immune protection against pathogens.

The molecular mechanism for how CD4^+^ T cells protect against CVB3 is unclear, but sex contributes to CD4^+^ T cell effector function during different diseases and viral infections (18, 20, 21, 50–53). CD4^+^ T cells differentiate into multiple effector functions, including Th1, Th2, Treg, and Tfh cell types, which impact viral infections. We hypothesize that increased activation of CD4^+^ T cells promotes Th1 polarization in female mice, leading to cell-mediated viral clearance. Our previous studies and others on the role of sex hormones on Th1 differentiation may support this hypothesis. First, we also recently found that CD8^+^ T cells protect against CVB3 infection in females, suggesting a strong Th1 response (30). Second, we previously observed an increase in the viral loads of multiple tissues including the heart, kidney, spleen and pancrease in male mice compared to female mice. Third, estrogen can help upregulate the Th1 transcription factor, T-box protein expressed in T cells (T-bet), in murine splenocytes (54). Finally, estradiol-treated mice have higher levels of IFN-γ producing CD4^+^ T cells, indicating the development of a Th1 response (55). Interestingly, *in vivo* data for CVB3 infections differs. Removing endogenous testosterone by gonadectomy promotes a Th2 response and increases Tregs following CVB3 infection (21). Furthermore, unlike many autoimmune diseases, autoimmune-induced myocarditis following CVB3 infection reflects a male bias driven by a Th1 response. Contrarily, Th2s in female mice reduce mortality (18, 24, 56). Therefore, while we have not fully characterized the CD4^+^ T cell subtypes following infection, it is possible that CVB3 also drives a predominantly Th2 response, limiting inflammation in our model. Thus, based on this dichotomy, it will be essential to characterize the CD4^+^ T cell effector subpopulations and their kinetics following infection of CVB3 in our mouse model.

Previous studies using C57BL/6 mice to evaluate the T cell response revealed a limited expansion of CD4^+^ and CD8^+^ T cells (19, 43, 44). However, these studies exclusively utilized male mice. Here, our data corroborate these findings in male mice but show a significant increase in CD4^+^ T cells in infected females, which further emphasizes the importance of sex differences in the immune response to pathogens. Further, our data demonstrating that CD4^+^ T cells are protective in female mice differ from previous models. Previous studies show that CD4^+^ T cells can be detrimental, rather than protective, to disease. For example, adult male CD4^+^ knockout mice are less susceptible to CVB3-induced disease (19). However, the discrepancy in our data may be due to strain-specific immune differences. Here, we used C57BL/6 mice that produce acute viral myocarditis, while Balb/c models develop autoimmune-mediated myocarditis following CVB3 infection. The contrasting results may be due to different predominant Th subtypes in Balb/c mice and C57BL/6 mice. Since the balance between Th1 and Th2 responses plays a role in exacerbating or limiting inflammation in the heart, this difference may be an underlying factor for our contrasting results.

Another possible explanation for the differences between our data and other labs is our model’s absence of type I IFN. We choose our model based on the ability of CVB3 to replicate in the intestine (26). Since this enteric virus is spread through the fecal-oral route, our model more closely mimics natural infection. However, we recognize the interaction between CD4^+^ T cells and type I IFN is complex. On the one hand, type I IFN can promote Th1 differentiation (57, 58). Conversely, they may also be required for the Tfh differentiation (59). Moreover, in the context of the same infection, the kinetics of type I IFN signaling can have opposing effects on CD4^+^ T cell fates (60). Interestingly, our previous data indicated that type I IFN signaling did not impact the CD8^+^ T cell response (30), but additional data is required to determine if the lack of type I IFNs could alter CD4^+^ T cell fates in male and female mice. Therefore, in the future, we plan to examine our findings in immune-competent wild-type C57BL/6 mice to understand how type I IFNs impact CD4^+^ T cells in the context of CVB3 infections.

Finally, the mechanism for how CD4^+^ T cells protect female mice is under investigation. Unfortunately, the cause of death in our mouse model is unknown. Unlike other mouse models for CVB3 that cause viral myocarditis, we currently do not have any data to suggest that myocarditis leads to mortality in our mice. However, we hypothesize that a classical model of Th1 T cells likely primes the CD8^+^ T cell response for cell-mediated killing of virally infected cells in target organs that limit mortality. Interestingly, the CD8^+^ T cell response, while important to CVB3 clearance, is limited in our model as compared to other viral infections. Therefore, it is intriguing to speculate that cytotoxic CD4^+^ T cells may also play a role in limiting virally infected cells. These cytotoxic CD4^+^ T cells have been shown to limit viral infections in an MHC II-dependent mechanism (48). In addition to cytotoxic CD4^+^ T cells, newly recruited CD4^+^ intraepithelial lymphocytes in the intestine have also recently been shown to limit gut infection of adenovirus (61). The impact of cytotoxic CD4^+^ T cells and intraepithelial lymphocytes on CVB3 is unclear, but future studies may define their role in limiting CVB3 pathogenesis.

In summary, our data indicates that CD4^+^ T cells play a protective role in CVB3-induced mortality and protect female mice. These data highlight the importance of sex-dependent T cell responses to pathogens. While the mechanism is still unclear on what drives this sex bias in T cell response, ultimately uncovering these mechanisms will have essential implications in vaccine development to limit CVB3 infections.

## Data availability statement

The raw data supporting the conclusions of this article will be made available by the authors, without undue reservation.

## Ethics statement

The animal studies were approved by Indiana University School of Medicine Institutional Animal Care and Use Committee (IACUC). The studies were conducted in accordance with the local legislation and institutional requirements. Written informed consent was obtained from the owners for the participation of their animals in this study.

## Author contributions

AP: Conceptualization, Data curation, Formal analysis, Investigation, Methodology, Writing – original draft, Writing – review & editing. AD: Conceptualization, Data curation, Formal analysis, Investigation, Writing – original draft, Writing – review & editing. SC: Conceptualization, Data curation, Investigation, Methodology, Writing – review & editing. CC: Data curation, Investigation, Methodology, Writing – review & editing. MR: Conceptualization, Data curation, Formal analysis, Supervision, Writing – review & editing. LS: Conceptualization, Data curation, Formal analysis, Supervision, Writing – review & editing. CR: Conceptualization, Data curation, Formal analysis, Funding acquisition, Project administration, Supervision, Writing – original draft, Writing – review & editing.

## References

[1] vom Steeg LG Klein SL. SeXX matters in infectious disease pathogenesis. PloS Pathog (2016) 12:e1005374. doi: 10.1371/journal.ppat.1005374 26891052 PMC4759457

[2] Markle JG Fish EN. SeXX matters in immunity. Trends Immunol (2014) 35:97–104. doi: 10.1016/j.it.2013.10.006 24239225

[3] Fischinger S Boudreau CM Butler AL Streeck H Alter G. Sex differences in vaccine-induced humoral immunity. Semin Immunopathol (2019) 41:239–49. doi: 10.1007/s00281-018-0726-5 PMC6373179 30547182

[4] Huang Z Chen B Liu X Li H Xie L Gao Y. Effects of sex and aging on the immune cell landscape as assessed by single-cell transcriptomic analysis. Proc Natl Acad Sci U.S.A. (2021) 118(33):e2023216118. doi: 10.1073/pnas.2023216118 PMC8379935 34385315

[5] Pujantell M Altfeld M. Consequences of sex differences in Type I IFN responses for the regulation of antiviral immunity. Front Immunol (2022) 13:986840. doi: 10.3389/fimmu.2022.986840 36189206 PMC9522975

[6] Dodd KC Menon M. Sex bias in lymphocytes: Implications for autoimmune diseases. Front Immunol (2022) 13:945762. doi: 10.3389/fimmu.2022.945762 36505451 PMC9730535

[7] Weinstein Y Ran S Segal S. Sex-associated differences in the regulation of immune responses controlled by the MHC of the mouse. J Immunol (1984) 132:656–61. doi: 10.4049/jimmunol.132.2.656 6228595

[8] Baker RE Mahmud AS Miller IF Rajeev M Rasambainarivo F Rice BL. Infectious disease in an era of global change. Nat Rev Microbiol (2022) 20:193–205. doi: 10.1038/s41579-021-00639-z 34646006 PMC8513385

[9] Gundamraj V Hasbun R. Viral meningitis and encephalitis: an update. Curr Opin Infect Dis (2023) 36:177–85. doi: 10.1097/QCO.0000000000000922 37093042

[10] Qiao X Liu X Wang Y Li Y Wang L Yang Q. Analysis of the epidemiological trends of enterovirus A in Asia and Europe. J Infect Chemother (2023) 29:316–21. doi: 10.1016/j.jiac.2022.12.006 36528275

[11] Brouwer L Moreni G Wolthers KC Pajkrt D. World-wide prevalence and genotype distribution of enteroviruses. Viruses (2021) 13(3):434. doi: 10.3390/v13030434 PMC7999254 33800518

[12] Badrinath A Bhatta S Kloc A. Persistent viral infections and their role in heart disease. Front Microbiol (2022) 13:1030440. doi: 10.3389/fmicb.2022.1030440 36504781 PMC9730422

[13] Lee BE Davies HD. Aseptic meningitis. Curr Opin Infect Dis (2007) 20:272–7. doi: 10.1097/QCO.0b013e3280ad4672 17471037

[14] Fairweather D Beetler DJ Musigk N Heidecker B Lyle MA Cooper LT Jr.. Sex and gender differences in myocarditis and dilated cardiomyopathy: An update. Front Cardiovasc Med (2023) 10:1129348. doi: 10.3389/fcvm.2023.1129348 36937911 PMC10017519

[15] Horwitz MS La Cava A Fine C Rodriguez E Ilic A Sarvetnick N. Pancreatic expression of interferon-gamma protects mice from lethal coxsackievirus B3 infection and subsequent myocarditis. Nat Med (2000) 6:693–7. doi: 10.1038/76277 10835688

[16] Fairweather D Rose NR. Coxsackievirus-induced myocarditis in mice: a model of autoimmune disease for studying immunotoxicity. Methods (2007) 41:118–22. doi: 10.1016/j.ymeth.2006.07.009 PMC1764911 17161308

[17] Slifka MK Pagarigan R Mena I Feuer R Whitton JL. Using recombinant coxsackievirus B3 to evaluate the induction and protective efficacy of CD8+ T cells during picornavirus infection. J Virol (2001) 75:2377–87. doi: 10.1128/JVI.75.5.2377-2387.2001 PMC114821 11160741

[18] Huber SA Pfaeffle B. Differential Th1 and Th2 cell responses in male and female BALB/c mice infected with coxsackievirus group B type 3. J Virol (1994) 68:5126–32. doi: 10.1128/jvi.68.8.5126-5132.1994 PMC236456 8035512

[19] Henke A Huber S Stelzner A Whitton JL. The role of CD8+ T lymphocytes in coxsackievirus B3-induced myocarditis. J Virol (1995) 69:6720–8. doi: 10.1128/jvi.69.11.6720-6728.1995 PMC189582 7474082

[20] Huber SA Kupperman J Newell MK. Hormonal regulation of CD4(+) T-cell responses in coxsackievirus B3-induced myocarditis in mice. J Virol (1999) 73:4689–95. doi: 10.1128/JVI.73.6.4689-4695.1999 PMC112510 10233928

[21] Frisancho-Kiss S Coronado MJ Frisancho JA Lau VM Rose NR Klein SL. Gonadectomy of male BALB/c mice increases Tim-3(+) alternatively activated M2 macrophages, Tim-3(+) T cells, Th2 cells and Treg in the heart during acute coxsackievirus-induced myocarditis. Brain Behav Immun (2009) 23:649–57. doi: 10.1016/j.bbi.2008.12.002 PMC3148833 19126426

[22] Huber SA Sartini D Exley M. Vgamma4(+) T cells promote autoimmune CD8(+) cytolytic T-lymphocyte activation in coxsackievirus B3-induced myocarditis in mice: role for CD4(+) Th1 cells. J Virol (2002) 76:10785–90. doi: 10.1128/JVI.76.21.10785-10790.2002 PMC136647 12368321

[23] Huber SA Moraska A Choate M. T cells expressing the gamma delta T-cell receptor potentiate coxsackievirus B3-induced myocarditis. J Virol (1992) 66:6541–6. doi: 10.1128/jvi.66.11.6541-6546.1992 PMC240148 1328680

[24] Huber S Shi C Budd RC. Gammadelta T cells promote a Th1 response during coxsackievirus B3 infection *in vivo*: role of Fas and Fas ligand. J Virol (2002) 76:6487–94. doi: 10.1128/JVI.76.13.6487-6494.2002 PMC136276 12050361

[25] Huber SA Stone JE Wagner DH Jr. Kupperman J Pfeiffer L David C. gamma delta+ T cells regulate major histocompatibility complex class II(IA and IE)-dependent susceptibility to coxsackievirus B3-induced autoimmune myocarditis. J Virol (1999) 73:5630–6. doi: 10.1128/JVI.73.7.5630-5636.1999 PMC112621 10364312

[26] Robinson CM Wang Y Pfeiffer JK. Sex-dependent intestinal replication of an enteric virus. J Virol (2017) 91(7):e02101-16. doi: 10.1128/JVI.02101-16 PMC5355612 28100612

[27] Dhalech AH Corn CM Mangale V Syed F Condotta SA Richer MJ. Testosterone promotes the intestinal replication and dissemination of coxsackievirus B3 in an oral inoculation mouse model. J Virol (2022) 96:e0123222. doi: 10.1128/jvi.01232-22 36037480 PMC9472648

[28] Huber SA Job LP Auld KR. Influence of sex hormones on Coxsackie B-3 virus infection in Balb/c mice. Cell Immunol (1982) 67:173–9. doi: 10.1016/0008-8749(82)90210-6 6280880

[29] Lyden DC Olszewski J Feran M Job LP Huber SA. Coxsackievirus B-3-induced myocarditis. Effect of sex steroids on viremia and infectivity of cardiocytes. Am J Pathol (1987) 126:432–8. PMC1899641 3030117

[30] Dhalech AH Condotta SA Pattnaik A Corn C Richer MJ Robinson CM. Coxsackievirus B3 elicits a sex-specific CD8+ T cell response which protects female mice. PloS Pathog (2023) 19:e1011465. doi: 10.1371/journal.ppat.1011465 37669302 PMC10503745

[31] Ida-Hosonuma M Iwasaki T Yoshikawa T Nagata N Sato Y Sata T. The alpha/beta interferon response controls tissue tropism and pathogenicity of poliovirus. J Virol (2005) 79:4460–9. doi: 10.1128/JVI.79.7.4460-4469.2005 PMC1061561 15767446

[32] Ohka S Igarashi H Nagata N Sakai M Koike S Nochi T. Establishment of a poliovirus oral infection system in human poliovirus receptor-expressing transgenic mice that are deficient in alpha/beta interferon receptor. J Virol (2007) 81:7902–12. doi: 10.1128/JVI.02675-06 PMC1951287 17507470

[33] Oxenius A Bachmann MF Zinkernagel RM Hengartner H. Virus-specific MHC-class II-restricted TCR-transgenic mice: effects on humoral and cellular immune responses after viral infection. Eur J Immunol (1998) 28:390–400. doi: 10.1002/(SICI)1521-4141(199801)28:01<390::AID-IMMU390>3.0.CO;2-O 9485218

[34] Rai D Pham NL Harty JT Badovinac VP. Tracking the total CD8 T cell response to infection reveals substantial discordance in magnitude and kinetics between inbred and outbred hosts. J Immunol (2009) 183:7672–81. doi: 10.4049/jimmunol.0902874 PMC2808048 19933864

[35] Schlub TE Badovinac VP Sabel JT Harty JT Davenport MP. Predicting CD62L expression during the CD8+ T-cell response *in vivo* . Immunol Cell Biol (2010) 88:157–64. doi: 10.1038/icb.2009.80 PMC2824781 19859082

[36] Nolz JC Starbeck-Miller GR Harty JT. Naive, effector and memory CD8 T-cell trafficking: parallels and distinctions. Immunotherapy (2011) 3:1223–33. doi: 10.2217/imt.11.100 PMC3214994 21995573

[37] Chao CC Jensen R Dailey MO. Mechanisms of L-selectin regulation by activated T cells. J Immunol (1997) 159:1686–94. doi: 10.4049/jimmunol.159.4.1686 9257829

[38] McDermott DS Varga SM. Quantifying antigen-specific CD4 T cells during a viral infection: CD4 T cell responses are larger than we think. J Immunol (2011) 187:5568–76. doi: 10.4049/jimmunol.1102104 PMC3221938 22043009

[39] Butler NS Schmidt NW Vaughan AM Aly AS Kappe SH Harty JT. Superior antimalarial immunity after vaccination with late liver stage-arresting genetically attenuated parasites. Cell Host Microbe (2011) 9:451–62. doi: 10.1016/j.chom.2011.05.008 PMC3117254 21669394

[40] Butler NS Moebius J Pewe LL Traore B Doumbo OK Tygrett LT. Therapeutic blockade of PD-L1 and LAG-3 rapidly clears established blood-stage Plasmodium infection. Nat Immunol (2011) 13:188–95. doi: 10.1038/ni.2180 PMC3262959 22157630

[41] Kimura D Miyakoda M Kimura K Honma K Hara H Yoshida H. Interleukin-27-producing CD4(+) T cells regulate protective immunity during malaria parasite infection. Immunity (2016) 44:672–82. doi: 10.1016/j.immuni.2016.02.011 26968425

[42] Pardy RD Rajah MM Condotta SA Taylor NG Sagan SM Richer MJ. Analysis of the T cell response to zika virus and identification of a novel CD8+ T cell epitope in immunocompetent mice. PloS Pathog (2017) 13:e1006184. doi: 10.1371/journal.ppat.1006184 28231312 PMC5322871

[43] Kemball CC Harkins S Whitmire JK Flynn CT Feuer R Whitton JL. Coxsackievirus B3 inhibits antigen presentation *in vivo*, exerting a profound and selective effect on the MHC class I pathway. PloS Pathog (2009) 5:e1000618. doi: 10.1371/journal.ppat.1000618 19834548 PMC2757675

[44] Kemball CC Harkins S Whitton JL. Enumeration and functional evaluation of virus-specific CD4+ and CD8+ T cells in lymphoid and peripheral sites of coxsackievirus B3 infection. J Virol (2008) 82:4331–42. doi: 10.1128/JVI.02639-07 PMC2293035 18305030

[45] Christiaansen AF Dixit UG Coler RN Marie Beckmann A Reed SG Winokur PL. CD11a and CD49d enhance the detection of antigen-specific T cells following human vaccination. Vaccine (2017) 35:4255–61. doi: 10.1016/j.vaccine.2017.06.013 PMC5551405 28662951

[46] Bohmer RM Bandala-Sanchez E Harrison LC. Forward light scatter is a simple measure of T-cell activation and proliferation but is not universally suited for doublet discrimination. Cytometry A (2011) 79:646–52. doi: 10.1002/cyto.a.21096 21695774

[47] Oshimi K. Clinical features, pathogenesis, and treatment of large granular lymphocyte leukemias. Intern Med (2017) 56:1759–69. doi: 10.2169/internalmedicine.56.8881 PMC5548667 28717070

[48] Snell LM Xu W Abd-Rabbo D Boukhaled G Guo M Macleod BL. Dynamic CD4(+) T cell heterogeneity defines subset-specific suppression and PD-L1-blockade-driven functional restoration in chronic infection. Nat Immunol (2021) 22:1524–37. doi: 10.1038/s41590-021-01060-7 PMC10286806 34795443

[49] Snell LM Osokine I Yamada DH de la Fuente JR Elsaesser HJ Brooks DG. Overcoming CD4 th1 cell fate restrictions to sustain antiviral CD8 T cells and control persistent virus infection. Cell Rep (2016) 16:3286–96. doi: 10.1016/j.celrep.2016.08.065 PMC5669380 27653690

[50] Umair M Fazazi MR Rangachari M. Biological sex as a critical variable in CD4(+) effector T cell function in preclinical models of multiple sclerosis. Antioxid Redox Signal (2022) 37:135–49. doi: 10.1089/ars.2021.0202 PMC9293683 34538129

[51] Ahnstedt H McCullough LD. The impact of sex and age on T cell immunity and ischemic stroke outcomes. Cell Immunol (2019) 345:103960. doi: 10.1016/j.cellimm.2019.103960 31519365 PMC6832888

[52] Huber SA. Coxsackievirus B3-induced myocarditis: infection of females during the estrus phase of the ovarian cycle leads to activation of T regulatory cells. Virology (2008) 378:292–8. doi: 10.1016/j.virol.2008.05.015 PMC2596296 18586295

[53] El-Badry E Macharia G Claiborne D Brooks K Dilernia DA Goepfert P. Better Viral Control despite Higher CD4(+) T Cell Activation during Acute HIV-1 Infection in Zambian Women Is Linked to the Sex Hormone Estradiol. J Virol (2020) 94(16):e00758-20. doi: 10.1128/JVI.00758-20 PMC7394904 32461316

[54] Karpuzoglu E Phillips RA Gogal RM Jr. Ansar Ahmed S. IFN-gamma-inducing transcription factor, T-bet is upregulated by estrogen in murine splenocytes: role of IL-27 but not IL-12. Mol Immunol (2007) 44:1808–14. doi: 10.1016/j.molimm.2006.08.005 PMC3097111 17046061

[55] Maret A Coudert JD Garidou L Foucras G Gourdy P Krust A. Estradiol enhances primary antigen-specific CD4 T cell responses and Th1 development *in vivo.* Essential role of estrogen receptor alpha expression in hematopoietic cells. Eur J Immunol (2003) 33:512–21. doi: 10.1002/immu.200310027 12645950

[56] Abston ED Coronado MJ Bucek A Bedja D Shin J Kim JB. Th2 regulation of viral myocarditis in mice: different roles for TLR3 versus TRIF in progression to chronic disease. Clin Dev Immunol (2012) 2012:129486. doi: 10.1155/2012/129486 22013485 PMC3195533

[57] Way SS Havenar-Daughton C Kolumam GA Orgun NN Murali-Krishna K. IL-12 and type-I IFN synergize for IFN-gamma production by CD4 T cells, whereas neither are required for IFN-gamma production by CD8 T cells after Listeria monocytogenes infection. J Immunol (2007) 178:4498–505. doi: 10.4049/jimmunol.178.7.4498 PMC2626161 17372008

[58] Ray JP Marshall HD Laidlaw BJ Staron MM Kaech SM Craft J. Transcription factor STAT3 and type I interferons are corepressive insulators for differentiation of follicular helper and T helper 1 cells. Immunity (2014) 40:367–77. doi: 10.1016/j.immuni.2014.02.005 PMC3992517 24631156

[59] Nakayamada S Poholek AC Lu KT Takahashi H Kato M Iwata S. Type I IFN induces binding of STAT1 to Bcl6: divergent roles of STAT family transcription factors in the T follicular helper cell genetic program. J Immunol (2014) 192:2156–66. doi: 10.4049/jimmunol.1300675 PMC3967131 24489092

[60] Osokine I Snell LM Cunningham CR Yamada DH Wilson EB Elsaesser HJ. Type I interferon suppresses *de novo* virus-specific CD4 Th1 immunity during an established persistent viral infection. Proc Natl Acad Sci U.S.A. (2014) 111:7409–14. doi: 10.1073/pnas.1401662111 PMC4034239 24799699

[61] Parsa R London M Rezende de Castro TB Reis B Buissant des Amorie J Smith JG. Newly recruited intraepithelial Ly6A(+)CCR9(+)CD4(+) T cells protect against enteric viral infection. Immunity (2022) 55(7):1234-1249.e6. doi: 10.1016/j.immuni.2022.05.001 PMC9283333 35617965

